# Ibrutinib-associated atrial fibrillation treatment with catheter ablation

**DOI:** 10.1016/j.hrcr.2021.08.003

**Published:** 2021-11-15

**Authors:** Ridhima Kapoor, Muhammad Fazal, Paul Cheng, Ronald Witteles, June-Wha Rhee, Tina Baykaner

**Affiliations:** Department of Medicine, Stanford University School of Medicine, Stanford, California

**Keywords:** Atrial fibrillation, Catheter ablation, Ibrutinib, Lymphoma, Tyrosine kinase inhibitors

**A Case for Education Quiz**

**Test your knowledge!**

Take an interactive quiz related to this article: https://www.heartrhythmcasereports.com/content/quiz_archive

## Introduction

**Figure 1:**
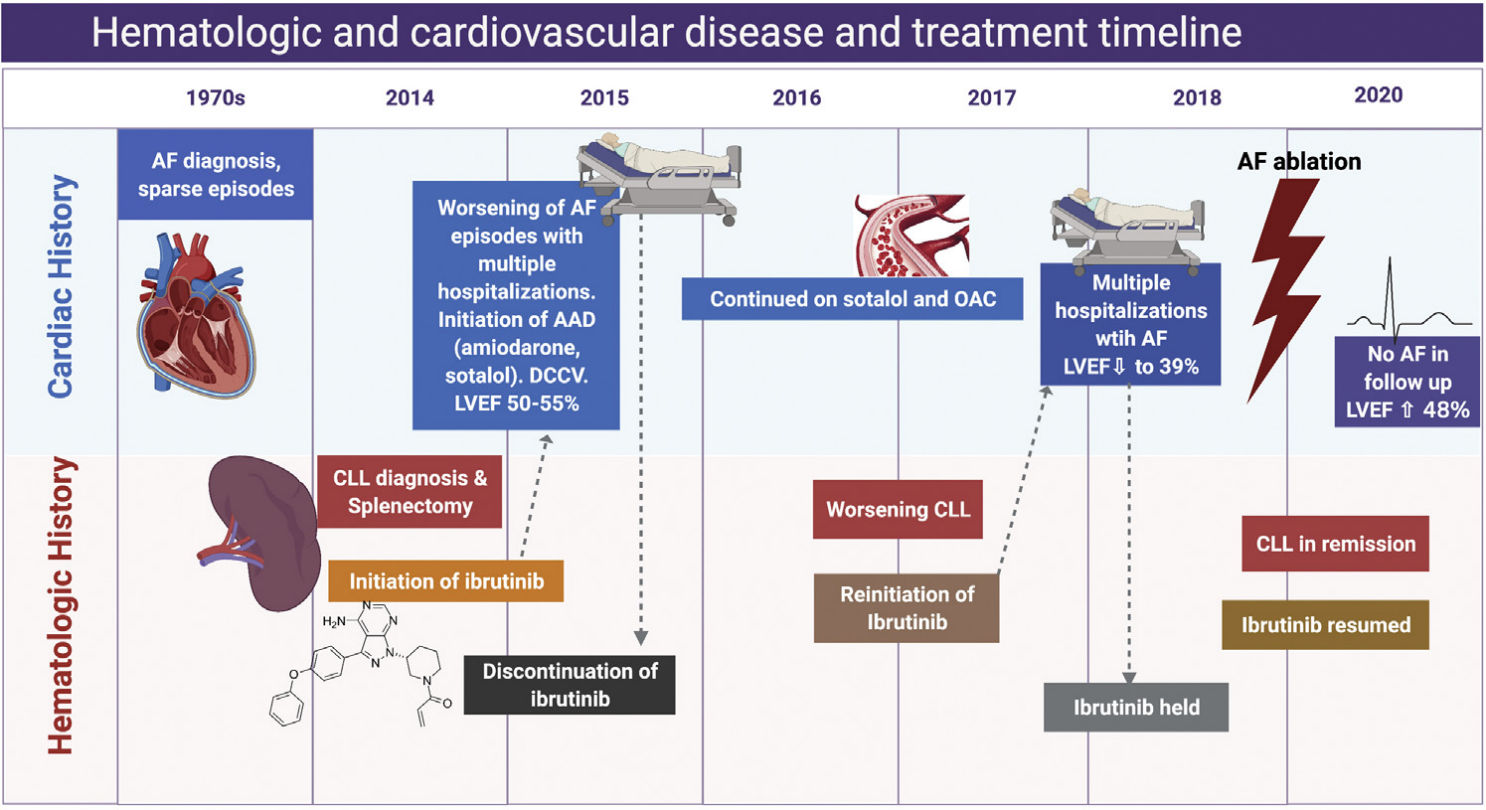
Timeline showing cardiovascular and hematologic disease and treatment course. AF = atrial fibrillation; CLL = chronic lymphocytic leukemia; AAD = antiarrhythmic drug; OAC = oral anticoagulant. (Figure created with BioRender.com.)

Tyrosine kinase inhibitors (TKIs) have been widely used to treat various malignancies, dramatically improving survival. The use of these treatments has been limited by adverse effects. Ibrutinib, a Bruton’s tyrosine kinase (BTK) inhibitor, has been associated with increased prevalence of atrial fibrillation (AF) in approximately 5%–16% of patients, especially those with prior history of AF,^1^ and often leads to treatment discontinuation in a significant proportion of patients. Management of AF in this patient population also presents a unique challenge. Current guidelines recommend rate control as the primary strategy,^2^^,^^3^ which can be challenging, since pharmacological management is limited by significant drug interactions with cancer therapies as well as potential side effects. Additionally, the need for anticoagulation in this strategy is challenged by increased risk of bleeding owing to inhibition of collagen-induced platelet aggregation by ibrutinib.^4^ Recent studies document the long-term benefits of catheter ablation in maintaining sinus rhythm in the general population. We present a case of ibrutinib-associated symptomatic AF, which was successfully treated with ablation therapy, and allowed the patient to return to ibrutinib therapy for adequate management of his cancer (Figure 1).

## Case report

### Initial presentation

We describe a 70-year-old man who was diagnosed with stage IV B-cell chronic lymphocytic lymphoma (CLL) at the age of 65. At the time of diagnosis, he had abnormal lymphocytosis and massive splenomegaly, consistent with Rai stage II with extranodal involvement. He underwent splenectomy, with pathology consistent with CLL. Fluorescence in situ hybridization studies were positive for deletion in 13q. He was subsequently started on ibrutinib for the treatment of CLL.

While the patient had a remote history of rare paroxysms of AF in the setting of acute illness such as viral infection and idiopathic myocarditis, he had never required long-term rate-controlling, antiarrhythmic, or anticoagulation therapy. However, shortly after the initiation of ibrutinib, he developed symptomatic, persistent AF and was started on amiodarone. Despite this, he was repeatedly hospitalized for symptomatic AF requiring cardioversion, for which he was started on digoxin and apixaban while continuing the amiodarone. He then developed progressive dyspnea on exertion, for which an echocardiogram was obtained, showing preserved left ventricular (LV) function with ejection fraction (EF) of 50%–55%. Given his persistent shortness of breath, he underwent a series of diagnostic tests, including a bronchoscopy, chest computed tomography, and lung biopsy, and was eventually diagnosed with idiopathic pulmonary fibrosis, which was attributed to amiodarone use in the background of CLL. Subsequently, he was started on corticosteroid therapy. Amiodarone was discontinued and instead sotalol therapy was initiated for rhythm control. Ibrutinib was also discontinued owing to uncontrolled AF during this time. With these measures, his cardiopulmonary condition gradually improved. He did not require any further hospitalizations or interventions for AF and remained in sinus rhythm.

Three years later, the patient’s CLL progressed with symptomatic anemia and thrombocytopenia, for which he was restarted on ibrutinib. Shortly after the reinitiation of ibrutinib therapy, he developed recurrent persistent AF with rapid ventricular response, resulting in hospital admission, where he underwent cardioversion. Sotalol was discontinued owing to ineffectiveness and instead he was started on dofetilide. He also developed new moderate LV dysfunction with EF of 39%, owing to suspected tachycardia-mediated cardiomyopathy. He was subsequently started on guideline-directed heart failure therapies. Digoxin and beta-blockade therapies were not adequate in achieving rate control during AF. Calcium channel blockade was not considered owing to significant drug-drug interaction with ibrutinib. Again, amiodarone was not considered for his underlying history of idiopathic pulmonary fibrosis. At this point, given inability to achieve rate or rhythm control with pharmacologic approach, the patient was considered for catheter ablation for rhythm control strategy. Of note, the patient did not have any ventricular arrhythmias or conduction abnormalities.

In terms of anticoagulation and stroke risk, the patient had a CHA_2_DS_2_-VASc score of 4 and was started on anticoagulation after increased prevalence of AF episodes after initiation of ibrutinib. He was initially on apixaban and then switched to rivaroxaban owing to patient preference of once-a-day dosing. He remained on anticoagulation with no adverse effects or significant bleeding.

### Catheter ablation therapy and outcomes

He presented in AF with ventricular rates ranging from 110 to 120 beats per minute on the day of the procedure. Under general anesthesia, transesophageal echocardiogram was performed with no evidence of left atrial appendage thrombus and peak left atrial appendage emptying velocity of 56 cm/s. Biatrial electroanatomic reconstruction was done with the aid of CARTO (Biosense Webster, Irvine, CA) 3-dimensional mapping and intracardiac echocardiography. Baseline activated clotting time (ACT) was 104 seconds. Intravenous heparin was administered during the procedure to maintain ACT greater than 300 seconds. During the procedure ACT ranged from 303 to 369 seconds after transseptal access was obtained.

**Figure 2:**
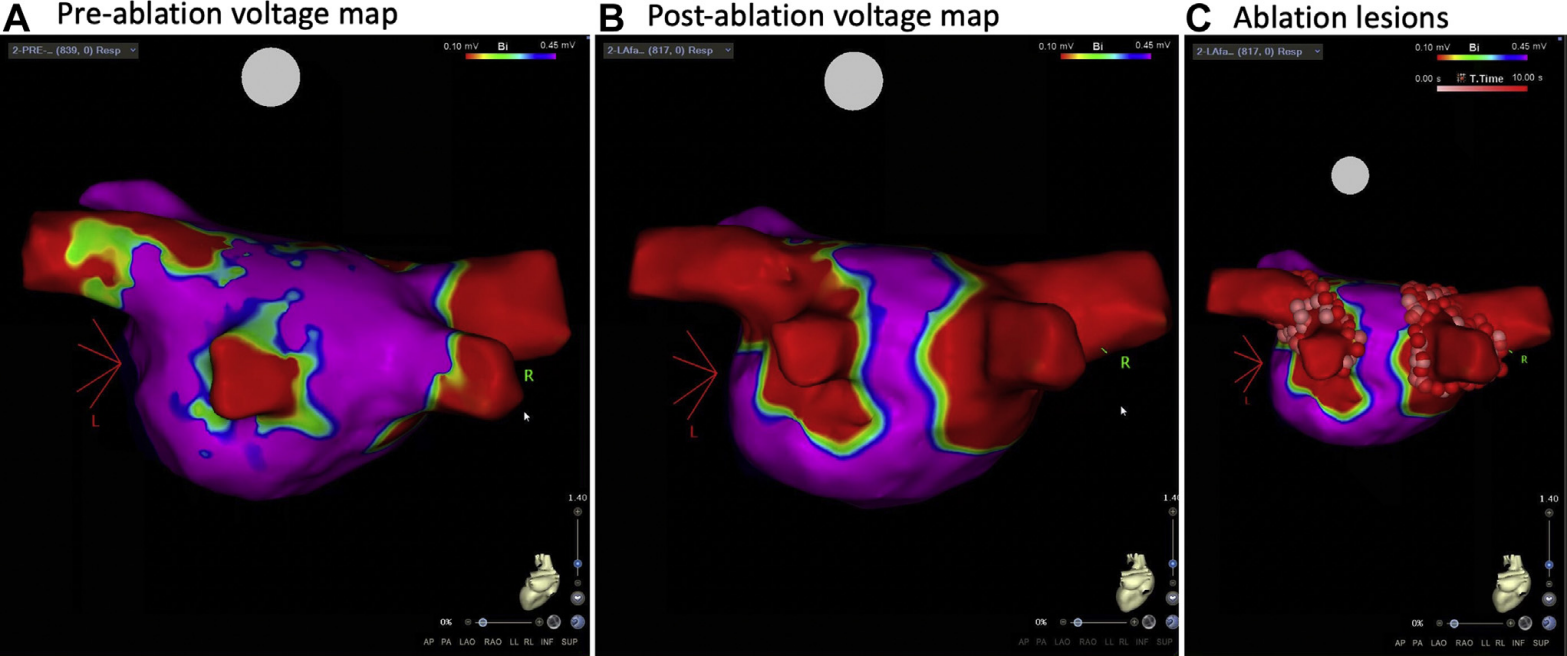
Electroanatomic maps of the left atrium in posteroanterior view showing voltage in color scale where purple depicts healthy atrial tissue (electrogram amplitude >0.45 mV) and red depicts scar tissue (electrogram amplitude <0.10 mV) preablation (**A**) and postablation (**B**) with ablation lesions (**C**) showing wide-area circumferential ablation and carina lines to achieve pulmonary vein isolation.

Baseline biatrial voltage mapping was performed with a multipolar catheter (PentaRay; Biosense Webster) showing no significant low-voltage areas while in AF (Figure 2A). Two left pulmonary veins and 2 right pulmonary veins were identified. Radiofrequency ablation was performed with an irrigated-tip contact force–sensing catheter with temperature control (SmartTouch STSF; Biosense Webster). Wide area circumferential ablation and carinal ablation were performed with point-by-point lesions to achieve pulmonary vein isolation. Lesion duration was guided by signal reduction and impedance trends. Total ablation time was 1733 seconds with average power of 30 watts.

Following ablation, he was externally cardioverted to assess for pulmonary vein entrance and exit block. Postablation voltage mapping was performed in sinus rhythm and demonstrated entrance block to the pulmonary veins (Figure 2B and 2C). Pulmonary vein exit block was confirmed with pacing within each vein with a multipolar mapping catheter. Although additional mapping, ablation of lines, or posterior wall isolation are commonly performed in patients with persistent AF; given the relatively acute worsening of AF episodes in our patient in the setting of ibrutinib initiation, we elected to limit his initial ablation procedure to pulmonary vein isolation only. Following ablation, he remained free of AF and was able to resume ibrutinib treatment. No recurrence of AF was noted on a 14-day cardiac event monitor at 18-month follow-up. Transthoracic echocardiogram 6 months postablation demonstrated improvement in LVEF from 39% to 48% and a decrease in left atrial indexed volume from 61.4 mL/m^2^ to 36.3 mL/m^2^.

## Discussion

Ibrutinib inhibits BTK, an essential enzyme for signaling pathways important for B-cell survival and proliferation, which is often upregulated in CLL and other B-cell malignancies. Inhibition of BTK by ibrutinib has resulted in remarkable tumor suppression and higher rates of disease remission. Unfortunately, ibrutinib use is associated with a 5-fold increased risk of development of new AF.^5^ Incessant AF can limit treatment options, and AF was the primary reason for discontinuation of ibrutinib therapy in 56% of patients in one study.^5^ The mechanism of AF in TKI therapy has been linked to calcium dysregulation^6^ and inhibition of cardiac phosphoinositide 3-kinase–Akt signaling,^7^ but remains poorly understood. A recent study showed that off-target inhibition of C-terminal Src kinase, a nonreceptor tyrosine kinase that inactivates Src kinase family members, may be responsible for the increased atrial arrhythmogenicity seen with ibrutinib administration.^8^ Overall, it is postulated that TKI treatment may lower the threshold of inducible AF, hence increasing the development of de novo AF as well as increasing the burden of any pre-existing AF.

Cancer patients on TKIs face unique challenges in AF treatments. First, potential drug-drug interactions between TKI and AF therapies may limit available treatment options. Calcium channel blockers such as verapamil and diltiazem, commonly used for AF rate control, are inhibitors of CYP3A4, a primary site for ibrutinib metabolism, and are therefore avoided in management. The serum level of amiodarone, the most commonly used rhythm control agent for AF, may be increased by ibrutinib through inhibition of p-glycoprotein, potentially increasing the risk of side effects. Second, an increased bleeding risk limits the administration of appropriate anticoagulation therapies for stroke prevention. Given their poor candidacy for long-term oral anticoagulation (OAC) therapy with a coexisting bleeding diathesis, and potentially increased clotting risk owing to malignancy, patients suffer from a higher risk of stroke. In a TKI registry, all of the patients with AF who were indicated for OAC were prescribed OAC,^9^ but these patients had more bleeding events compared to control patients who were not receiving TKI therapy. The increased risk of bleeding could be due to ibrutinib-induced platelet aggregation defects as seen in some in vitro studies.^7^ Owing to this very reason of platelet dysfunction and subsequent increased bleeding risk (eg, gastrointestinal bleed), the HAS-BLED score was deemed not adequate when assessing bleeding risk in cancer patients. On the other hand, cancer itself induces hypercoagulability and the CHA2DS2-VASc score may underestimate the risk of stroke. Thus, comprehensive risk and benefit discussion needs to take place in shared decision-making regarding anticoagulation therapy. If OAC is indeed felt appropriate, data suggest overall safety and effectiveness of direct OAC therapy in patients with cancer and AF.^10^

In patients with AF, it is important to determine whether a rate control or rhythm control approach should be considered. This question has been the subject of multiple studies with conflicting results that may reflect the types of patients studied, the interventions used to achieve rhythm control, and the outcomes assessed.^11^ In general, the decision is based on the type and frequency of AF episodes, the symptoms the patient has, and any underlying comorbidities that can complicate therapy. There is emerging evidence on the safety of rhythm control as well as AF ablation compared to conservative management,^12^ with mortality benefit with AF ablation in certain subgroups of patients.^12^^,^^13^ Recent studies have also suggested benefits of early rhythm control therapy with antiarrhythmic drugs and/or AF ablation in reducing cardiovascular death, hospitalizations, and stroke.^14^ Despite accumulating evidence on the safety and efficacy of aggressive rhythm control strategies, there are no studies to date that systematically evaluated the role of catheter ablation of AF in cancer patients on ibrutinib or, more broadly, TKIs.

Ablation therapies may be considered early in the management of patients on TKI who develop AF. Successful ablation therapy can completely suppress underlying AF, thereby preventing subsequent need for extensive or chronic pharmacologic therapies for rhythm control, providing wider therapeutic options for cancer treatment. As shown in this case, our patient had an increasing burden of AF upon initiation of ibrutinib therapy, which remained poorly controlled despite antiarrhythmic (amiodarone, dofetilide, and sotalol) and rate-controlling (beta blockers and digoxin) therapies. He also had a drop in LVEF, which was likely contributed at least in part by his poorly controlled AF. Despite his long history of paroxysmal AF, albeit with only rare episodes pre–ibrutinib therapy, the patient underwent successful AF ablation therapy with no further episodes of AF >12 months postablation, allowing him to be reinitiated on ibrutinib therapy, with subsequent remission of his CLL. As seen in this case, in a subset of AF patients on TKIs with significant symptoms, competing comorbidities (eg, lung disease), or high-risk features for bleeding, ablation therapy may be considered as a safe and potentially definitive alternative therapy to pharmacologic interventions.

In summary, this case report demonstrates success of catheter ablation for AF after failed medical therapy, allowing for continuation of ibrutinib therapy. A procedural approach to patients on TKI with exacerbation of pre-existing AF or new-onset AF may prevent treatment interruption and/or discontinuation. Pulmonary vein isolation was performed in this patient, but the ideal approach for ablation in this patient population remains unclear. Further studies are needed to investigate the safety and efficacy of AF ablation as well as ideal ablation of strategy in treating TKI-associated AF.
